# Effect of Implant Design on Fracture Resistance, Mode, and Crystal Structure of One‐ and Two‐Piece Zirconia Implants With Ceramic or Titanium Abutment Screws: An In Vitro Study

**DOI:** 10.1111/clr.70149

**Published:** 2026-06-13

**Authors:** Abdulaziz Gul, Evaggelia Papia, Siamak Eqtesadi, Magnus Anderson, Jonas P. Becktor, Per Vult von Steyern

**Affiliations:** ^1^ Department of Materials Science and Technology, Division 2, Faculty of Odontology Malmö University Malmö Sweden; ^2^ Department of Oral and Maxillofacial Surgery and Oral Medicine, Division 2, Faculty of Odontology Malmö University Malmö Sweden; ^3^ Department of Oral and Maxillofacial Surgery, Faculty of Dental Medicine Umm Al‐Qura University Makkah Saudi Arabia; ^4^ The Nordic Institute of Dental Materials, NIOM Oslo Norway; ^5^ Department of Prosthetic Dentistry and Oral Function, Institute of Clinical Dentistry University of Oslo Oslo Norway

**Keywords:** bone‐level implants, dental implants, implant design, implant fracture, tissue‐level implants, zirconia implants

## Abstract

**Objectives:**

To evaluate the fracture resistance, fracture modes, and crystalline phase changes of zirconia one‐piece implants (OPI) and bone‐level (BLTPI) or tissue‐level, two‐piece implants (TLTPI), with ceramic (C) or titanium (T) abutment screws after dynamic preloading in a moist environment.

**Materials and Methods:**

Dynamic preloading under moist conditions and subsequent static load‐to‐fracture testing were performed to evaluate fracture resistance and fracture mode. Scanning electron microscopy (SEM) analysis of fracture surfaces was employed to supplement the observations of macroscopic fracture modes. Crystalline phase changes were assessed using micro‐Raman spectroscopy. Specimen preparations were performed in accordance with ISO 14801:2016. Five different ceramic (zirconia) implant designs were divided into the following groups: OPI, BLTPI (BLC, BLT), TLTPI (TLC, TLT). One‐way and two‐way ANOVA tests were used for comparisons and factor effect analysis.

**Results:**

Fracture resistance (N) and bending moment (N cm) differed significantly among the implant groups (*p* < 0.001), following the order TLTPI (775.2 ± 71 N) > OPI (605.7 ± 37 N) > BLTPI (498.3 ± 93 N). Within the two‐piece implants (TPI), tissue‐level designs (TL) outperformed bone‐level designs (BL), while designs with different abutment screw materials showed no significant difference (*p* = 0.982).

**Conclusions:**

Within the limitations of this in vitro study, the evaluated zirconia‐based OPI, BLTPI, and TLTPI systems demonstrate sufficient fracture resistance to support further clinical investigation. Implant‐level design (TL vs. BL) influences mechanical performance, whereas the influence of abutment screw material (ceramic vs. titanium) is less pronounced and varies according to the implant‐level design.

## 1 Introduction

Dental implants are widely used to replace missing teeth and restore oral function and esthetics by supporting a rehabilitation prosthesis. Although titanium alloy remains the gold standard for dental implant materials, increasing interest in metal‐free alternatives—particularly for patients with metal sensitivities—coupled with advancements in oxide ceramics, has expanded the use of zirconium dioxide (ZrO_2_, zirconia). Its favorable biocompatible mechanical properties and reported osseointegration and soft‐tissue responses have led to the use of yttria‐stabilized tetragonal zirconia polycrystal (Y‐TZP) as a material for dental implants (Bethke et al. 2020; Gahlert et al. 2007; Roehling et al. 2019). Furthermore, the martensitic toughening mechanism—characterized by stress‐induced transformation from the tetragonal (*t*) to the monoclinic (*m*) phase—enhances crack resistance by generating a volume increase at the crack tip (Garvie et al. 1990).

Previous studies have shown that low‐temperature degradation (LTD) or aging can affect the surface of zirconia under clinical use through *t*–*m* crystalline phase transformations and/or grain pull‐out, resulting in reduced material strength (Cattani‐Lorente et al. 2011; Chevalier et al. 2007). Dynamic preloading may generate localized tensile stresses at the surface and at stress concentrators, potentially activating or accelerating stress‐assisted *t*–*m* transformation. Under moist conditions, the combined action of water and repeated loading may enhance LTD‐related phase transformation. Accordingly, moist conditions, together with dynamic preloading, have been reported to induce LTD and increase the monoclinic phase content (Borchers et al. 2010; Kohorst et al. 2012).

An in vitro study reported reduced fracture strength of zirconia dental implants after dynamic preloading (5 million cycles), equivalent to 20 years of clinical use, whereas artificial aging by autoclave has been associated with increased mechanical strength (Kohal et al. 2011; Monzavi et al. 2020). However, the effects of LTD remain inconclusive. Some studies report no considerable effect of LTD or aging on the mechanical properties of zirconia dental implants (Papanagiotou et al. 2006; Zhang et al. 2020).

A systematic review of in vitro studies found that implant designs influence the fracture strength of zirconia implants, with the one‐piece implants (OPI) showing higher fracture strength than two‐piece implants (TPI) (Bethke et al. 2020). The most used zirconia dental implant design in clinical practice is the OPI design, consisting of a single monolithic unit combining the implant and the abutment. In addition, clinical studies report acceptable 5‐year survival rates of 92%–98% for one‐piece zirconia implants, comparable to titanium implants (Balmer et al. 2020; Kohal et al. 2020; Oliva et al. 2010; Pjetursson et al. 2012; Rodriguez et al. 2018; Roehling et al. 2018). However, the OPI design has notable limitations. It allows only a one‐stage placement without soft tissue closure during healing, an option that may be unsuitable in cases of reduced primary stability or when bone augmentation is required. Moreover, the inability to correct implant angulation makes placement a more technique‐sensitive procedure (Andreiotelli et al. 2009; Parel and Schow 2005). To overcome these limitations, two‐piece zirconia dental implants were developed in bone‐level (BL) and tissue‐level (TL) designs, where the implant and abutment are separate components, addressing the restrictions of the one‐piece design (Cionca et al. 2017; Kammermeier et al. 2016; Rosentritt et al. 2014). However, the major concern with the two‐piece zirconia implants is the implant‐abutment connection. The reduced cross‐sectional material thickness of the implant, combined with the brittle nature of zirconia and the susceptibility of the two‐piece systems to aging, may increase the risk of fracture (Bethke et al. 2020; Cionca et al. 2021; Osman and Swain 2015; Stimmelmayr et al. 2020; Zhang et al. 2022). Consequently, manufacturers have tried to minimize concerns regarding the implant‐abutment connection by modifying the design and introducing different connection types. These include screw‐retained abutments using different types of abutment screw materials (ceramic, carbon‐reinforced PEEK, and titanium), cement‐retained abutments, or combinations of both screw and cement‐retained abutments.

Clinical and in vitro studies have recommended further investigation of two‐piece zirconia implant designs (Bethke et al. 2020; Cionca et al. 2021; Kohal et al. 2009; Mohseni et al. 2023). A recent systematic review of clinical studies reported a 96% survival rate for zirconia TPIs with up to 9 years of follow‐up (Bazal‐Bonelli et al. 2025). Screw‐retained two‐piece zirconia implant designs featuring ceramic abutment screws and ceramic abutments are relatively new to the market. An in vitro study investigating bone‐level two‐piece implants (BLTPI) with ceramic and titanium abutment screws reported fracture resistance adequate for clinical use (Kohal et al. 2024).

Given the wide range of zirconia dental implant designs and connection materials, there is a need to evaluate their performance under aging induced by dynamic preloading in a moist environment in order to determine the mechanical outcomes of each design. Therefore, the aim of the study was to evaluate the fracture resistance, fracture mode, and crystalline phase changes of different zirconia implant designs—one‐piece and bone‐level or tissue‐level two‐piece implants with ceramic or titanium abutment screws—after aging under dynamic preloading in a moist environment.

Based on the aim, the null hypotheses were:

### 1.1 Null Hypotheses

There is no difference in the fracture resistance and fracture mode between one‐piece and bone‐level or tissue‐level two‐piece zirconia dental implant designs after aging by dynamic preloading in a moist environment.

There is no difference in fracture resistance or fracture mode following dynamic preloading in a moist environment between bone‐level and tissue‐level designs of two‐piece zirconia implants, nor between designs with abutment screws made of ceramic versus titanium.

Crystalline phases of zirconia implants are not influenced by aging (dynamic preloading in a moist environment) or by fracture testing, regardless of implant design.

## 2 Materials and Methods

### 2.1 Specimens

The sample size for the one‐ and two‐piece zirconia dental implant designs was determined through power analysis based on previous studies reporting effect sizes (0.965), with 80% power and a 0.05 level of significance. This resulted in 18 specimens allocated to each of the three main groups (a total of 54 specimens). In addition, one extra specimen for each design (in total five) was included for post‐preloading surface and crystalline phases analyses.

In total, 59 zirconia dental implants (4.0 mm diameter and 10 mm length; Z‐Systems, Oensingen, Switzerland) were divided into three main groups based on implant design: one‐piece, bone‐level two‐piece, and tissue‐level two‐piece. Each two‐piece group was further divided according to the type of implant‐abutment connection (ceramic or titanium abutment screw). In total, five implant designs/material combinations were evaluated in the present study:

- Group: One‐piece implant (OPI)
- Group: Bone‐level two‐piece implant (BLTPI)
  ○ (subgroup): Bone‐level two‐piece implant with ceramic abutment screw (BLC)
  ○ (subgroup): Bone‐level two‐piece implant with titanium abutment screw (BLT)
- Group: Tissue‐level two‐piece implant (TLTPI)
  ○ (subgroup): Tissue‐level two‐piece implant with ceramic abutment screw (TLC)
  ○ (subgroup): Tissue‐level two‐piece implant with titanium abutment screw (TLT)

**FIGURE 1:**
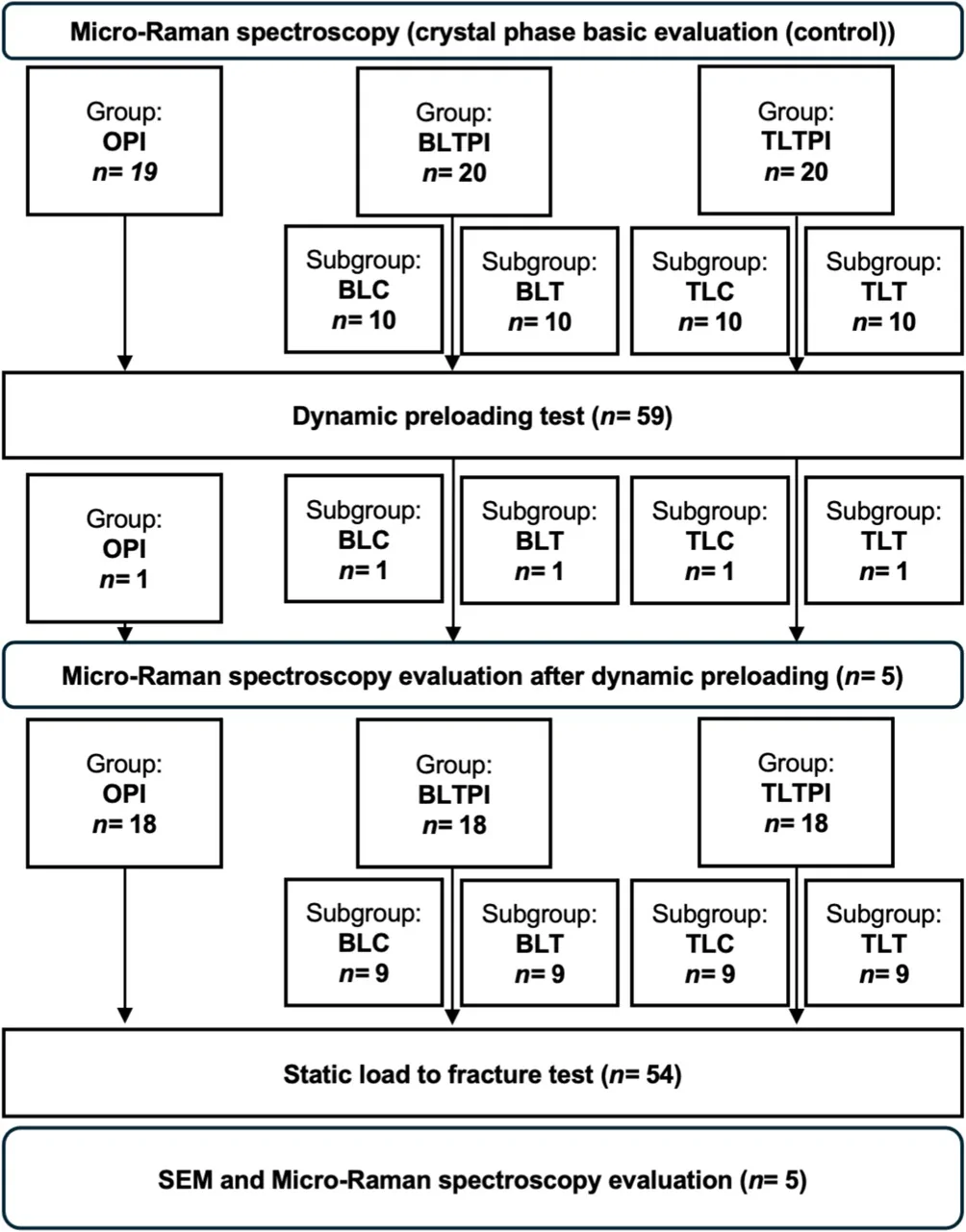
Study flow diagram. BL, bone‐level; BLC, bone‐level TPI with a ceramic screw; BLT, bone‐level TPI with a titanium screw; BLTPI, bone‐level two‐piece implant; *n*, number of specimens; OPI, one‐piece implant; SEM, scanning electron microscopy; TL, tissue‐level; TLC, tissue‐level TPI with a ceramic screw; TLT, tissue‐level TPI with a titanium screw; TLTPI, tissue‐level two‐piece implant; TPI, two‐piece implant.

**FIGURE 2:**
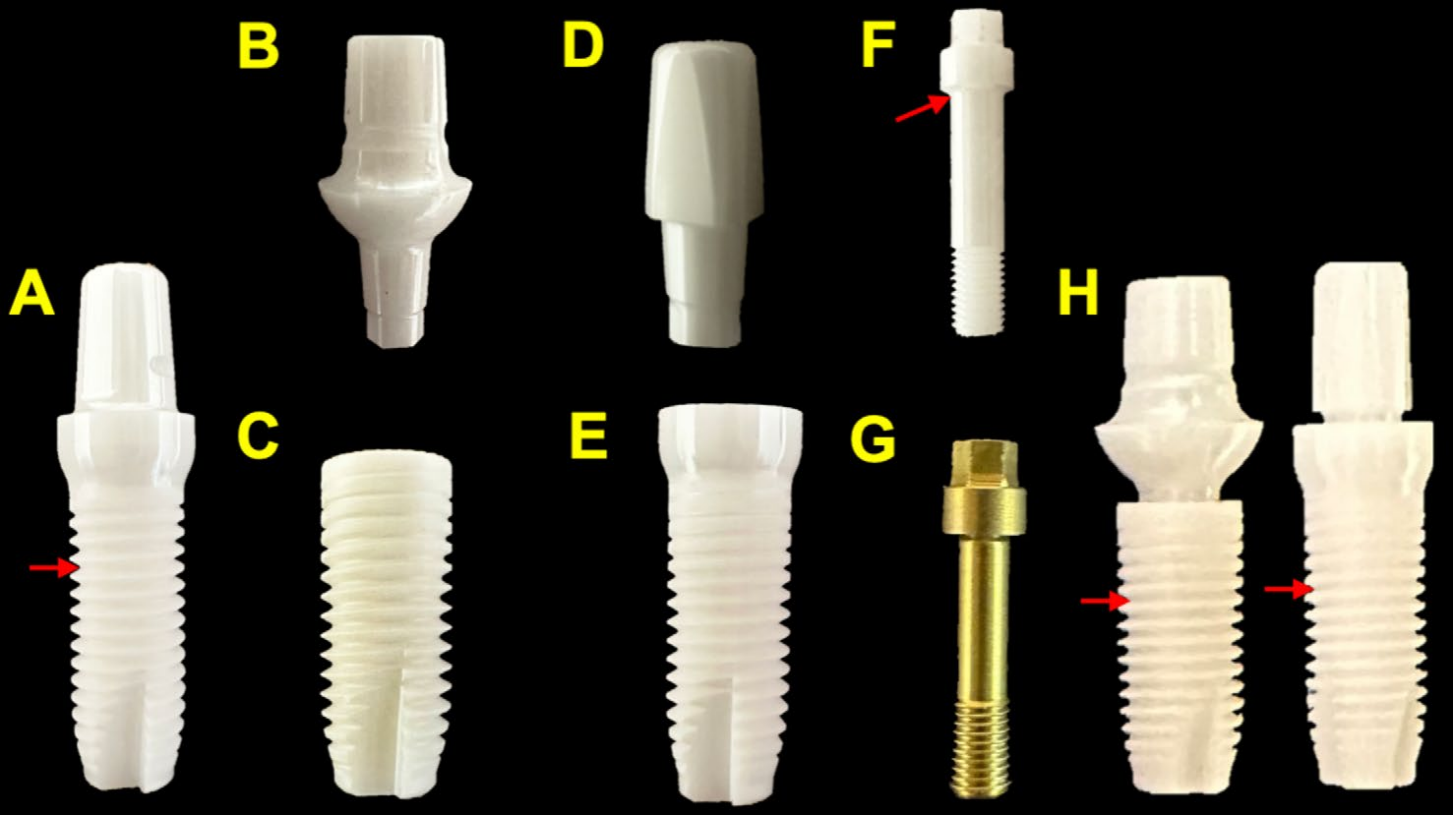
Components (product) of the implant–abutment systems used in the study. (A) One‐piece implant (Z5M, Z‐Systems, Switzerland). (B) Bone‐level abutment (BL‐A0025). (C) Bone‐level implant (Z5BL). (D) Tissue‐level abutment (TL‐A0055). (E) Tissue‐level implant (Z5TL). (F) Ceramic abutment screw (BL‐OSC‐H). (G) Titanium abutment screw (BL‐OST‐H). (H) Assembled bone‐level and tissue‐level two‐piece implants. Red arrows indicate sites selected for micro‐Raman evaluation of crystalline phases. These sites are located on the loading (tensile) side of the implants and ceramic abutment screw, where the oblique loading (30°) was applied.

See Figures 1 and 2, and Tables 1 and 2 for detailed information on the implant designs.

**TABLE 1:** Implant design geometry and specifications.

Main designs	One‐piece implant	Bone‐level two‐piece implant	Tissue‐level two‐piece implant
Manufacturer	Z‐Systems AG (Oensingen, Switzerland)		
Product	Z5M‐40‐10	Z5‐BL‐4010	Z5‐TL‐4010
Implant surface	Machined, followed by grit‐blasting (corundum) and laser modification (SLM; Z‐Systems) Surface roughness (Ra) 0.67 (grit‐blasted) 3 μm (grit‐blasted + SLM)		
Implant thread	V‐shaped thread with rounded edges		
Intraosseous diameter (mm)	4	4	4
Intraosseous length (mm)	10	10	10
Shoulder height (mm)	≈2.9	n/a	≈2.5
Straight abutment product	n/a	BL‐A0025	TL‐A0055
Abutment length (mm)	5	≈7.5	≈5.5
Abutment surface	Machined without further surface treatment		
Implant‐abutment connection	One‐piece	Screw retained two‐piece implant with internal conical connection. Depend on the friction fit and secured with either: Ceramic abutment screw M1.4 × 0.2 (14 N cm pretension) or Titanium abutment screw M1.4 × 02 (14 N cm pretension)	

Abbreviations: BL, bone‐level; TL, tissue‐level.

**TABLE 2:** Materials and mechanical properties of the components used in the experiment.

Material	Unit	Zirconia Y‐TZP^a^	Titanium grade 5^b^	Resin (Rigid 10K, Formlabs)^c^	Cobalt chromium (Remanium star, Dentaurum)^c^
Contents	—	ZrO_2_/Y_2_O_3_/Al_2_O_3_	Ti6AL/4V ELI	—	Co/Cr/W/Si
Composition	wt%	95/5/0.25	—	—	60.5/28/9/1.5
Processing	—	CIP, hot isostatic compaction, sintering, and HIP	—	—	—
Young's modulus	GPa	210	114	10	190
Density	g/cm^3^	6.05	—	—	—
Grain size	μm	< 0.35	—	—	—
Bending strength	MPa	≥ 1200	1054	—	845
Vickers hardness	Hv	1200	—	—	—
Fracture toughness K_1C_	MPa√m	8	—	—	—
Study components	—	Implant, abutments and abutment screw	Abutment screw	Specimen holder	Loading hemisphere or caps

Abbreviations: CIP, cold isostatic pressing; ELI, extra low interstitial; HIP, hot isostatic post‐compaction process; Y‐TZP, yttria‐stabilized tetragonal zirconia polycrystal.

^a^ Characteristics provided by the manufacturer (Z‐Systems).

^b^ Characteristics retrieved from de la Rosa Castolo et al. (2018).

^c^ Characteristics retrieved from the manufacturer's (Formlabs and Dentaurum) websites.

### 2.2 Preparation and Assembly

The implants, abutments, and abutment screws were used as supplied by the manufacturer.

The abutments were inserted into the implants and secured using the manufacturer‐provided screwdriver. According to the manufacturer's instructions, the abutment screws were tightened until the screwdriver tip broke or chipped off, corresponding to an applied torque of approximately 14 N cm.

All specimens were embedded in a resin specimen holder (Rigid 10K, Formlabs, Somerville, MA, USA) with a height of 20 mm and a width of 12 mm, produced using a Form 3B+ printer (Formlabs). The design incorporated a small notch to define the loading direction and a cylindrical hole (4 mm width, 7 mm depth) to guide implant positioning. A 4 mm‐diameter drill with marked depth indicators was used to ensure the specified embedding depth for each implant design group. The embedding depth was set according to ISO 14801:2016, positioning the implant's bone level 3 mm ± 0.5 mm above the top shoulder of the specimen holder to simulate bone loss in a clinical context (International Organization for Standardization 2016). The embedding resin has a modulus of elasticity of approximately 10 GPa, which meets the ISO 14801: 2016 recommendation of > 3 GPa.

**FIGURE 3:**
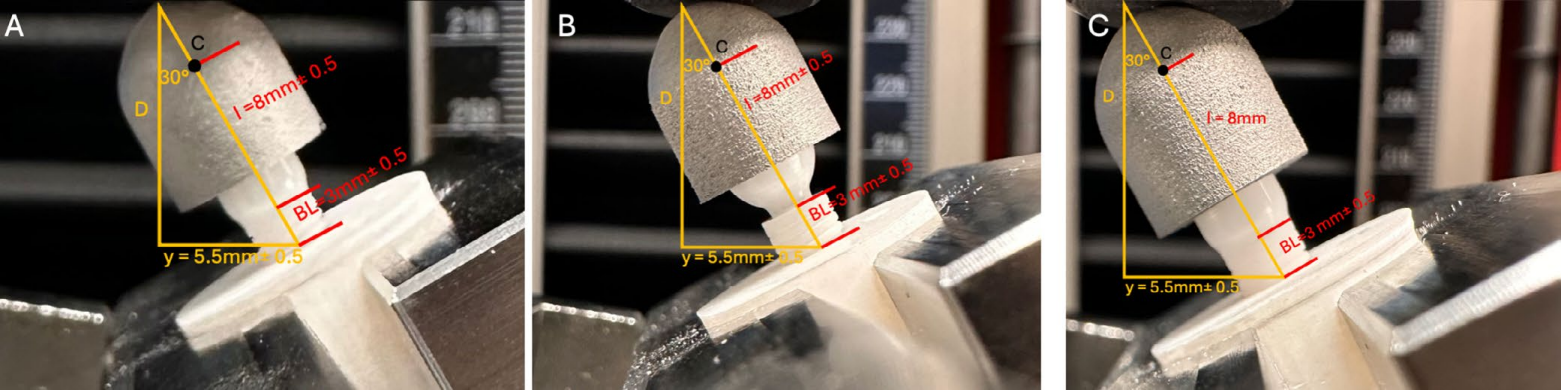
Specimen preparation with graphical measurement drawing in accordance with ISO 14801 recommendations, that is, external load direction (D) at a 30‐degrees angle to the implant axis, the implant position at a 3 mm offset or to simulate bone loss (BL) and a loading hemisphere designed with an 11 mm ± 0.5 distance from the center C of the loading hemisphere to the level of the specimen holder shoulder (I = 8 mm + BL = 3 mm = 11 ± 0.5) thereby the lever arm (y) is 5.5 mm ± 0.5. The embedding material has an elastic modulus of 10 GPa. (A) One‐piece implant (B) Bone‐level two‐piece implant (C) Tissue‐level two‐piece implant.

A Cobalt‐chromium loading hemisphere was designed using the stereolithography (STL) files of each abutment design provided by the manufacturer. The external (outer) design of the loading hemisphere had a height of 10.5 mm and a width of 9.5 mm. Following the ISO14801:2016 recommendation, the distance from the center of the loading hemisphere to the top of the specimen holder was set to 11 mm ± 0.5 mm for all specimen groups, and the load was applied at an angle of 30 (±2) degrees relative to the implant axis. Accordingly, the lever arm (y) was 5.5 mm ± 0.5. Three different loading‐hemisphere internal geometries were used, each designed to fit one of the three different types of implant abutment geometries (OPI, BLTPI, TLTPI). An approximately 40‐μm gap was provided to ensure a passive fit on the abutment surface. Details on the material characteristics and specimen preparation are provided in Table 2 and in Figure 3.

### 2.3 Dynamic Preloading Test

**FIGURE 4:**
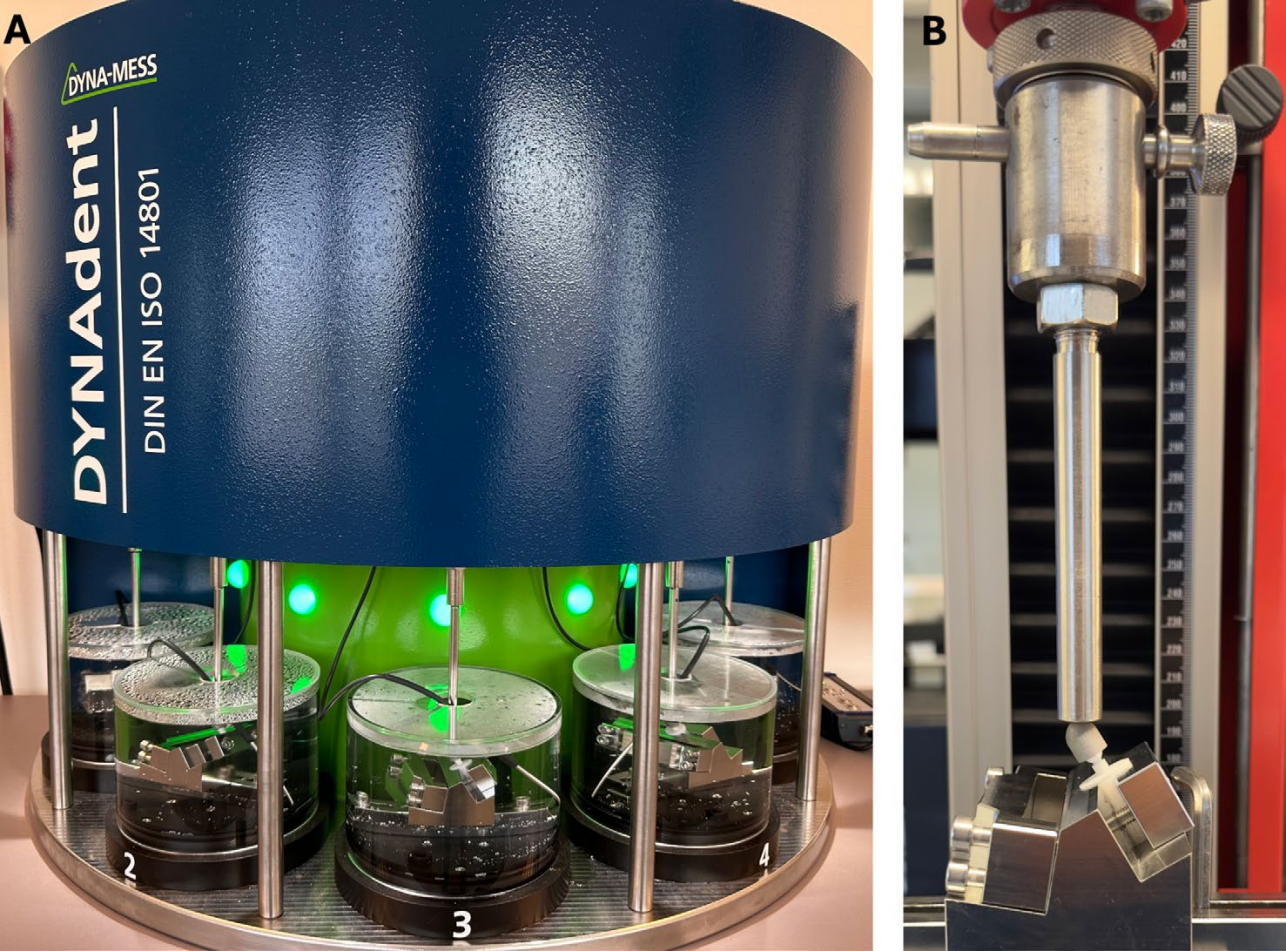
The machines and setup used in the dynamic preloading and static load‐to‐fracture tests. (A) Dynamic preloading machine (DYNAdent, DYNA‐MESS GmbH, Germany). (B) universal testing machine (ZwickiLine, Zwick–Roell, Ulm, Germany) for static load‐to‐fracture test.

All embedded specimens were mounted in a test jig at a loading angle of 30° ± 2°, in accordance with ISO 14801:2016 recommendations. Specimens were subjected to dynamic preloading for 2 million cycles using an ISO‐certified dynamic preloading machine (DYNAdent, DYNA‐MESS GmbH, Germany). A load of 100 N, equal to a bending moment of 55 N cm, was applied at a loading frequency of 2 Hz. Throughout the test, specimens were immersed in distilled water maintained at 37°C. Figure 4 shows the test setup. The specimens were visually checked once daily during the test. Any fracture, material yielding, deformation, or loosening of the implant‐abutment assembly was characterized as a failure to survive the dynamic preloading test.

### 2.4 Static Load‐to‐Fracture Test

After dynamic preloading, all surviving specimens were mounted in the same test jig used for the dynamic preloading test at a 30° ± 2° angle relative to the loading direction. A load was applied to the embedded implants until fracture using a universal testing machine (ZwickiLine, Zwick–Roell, Ulm, Germany) at a crosshead speed of 0.5 mm/min. The exact fracture resistance (*F*) in Newtons (N) was determined as the point where a sudden 20% drop in the loading curve occurred. Figure 4 shows the test setup. According to the ISO 14801: 2016, the bending moment (*M*, in N cm) was calculated using a 5.5 mm lever arm. For this test setup, the equation *M* = 5.5 × *F*/10 was applied (International Organization for Standardization 2016).

### 2.5 Fracture Mode

After the load‐to‐fracture test, fracture patterns were visually evaluated and supported with scanning electron microscopy (SEM) images for each zirconia implant design.

### 2.6 SEM of Fracture Surfaces

To complement the macroscopic fracture mode observations, representative fracture surfaces from the main implant design groups (OPI, BLTPI, and TLTPI) were examined using SEM.

SEM imaging was performed using a Hitachi TM4000 plus Tabletop (Hitachi High‐Tech Corporation, Tokyo, Japan) microscope with a range of 60‐5k magnifications using the detector for back scattering electrons.

### 2.7 Crystalline Phases

The evaluation sites for crystalline phases were determined based on the location of the high tensile stress concentration (the loading side of the implant) in the implant and the neck of the abutment screw for each design. These locations were identified following a finite element analysis (FEA) done by the same group of researchers for the present study (Gul et al. 2026). The selected sites are indicated by red arrows in Figure 2.

Micro‐Raman spectroscopy analysis (MonoVista CRS+, Spectroscopy & Imaging GmbH, Germany) was used to qualitatively evaluate the transition from *t* to *m* crystalline phases in one specimen from each group and subgroup before and after the dynamic preloading test, and after the load‐to‐fracture test. Each specimen was attached to a glass slide using double‐sided tape. Crystalline phase characterization was done using a 532 nm wavelength laser. Spectra were obtained with a 5 s integration time and 10 acquisitions to improve the *s*/*n* ratio, resulting in a total exposure time of 50 s per specimen. The results were presented as plotted graphs for each design.

The crystalline phases were estimated using the Raman‐based method proposed by (Katagiri 1988), which relates the relative intensities of characteristic *m* and *t* Raman bands. Where the intensities (peak heights) at approximately 178 and 188 cm^−1^ represent the intensity (peak height) of the *m* Raman band and at approximately 262 cm^−1^ represent the intensity (peak height) of the *t* Raman band. The monoclinic phase fraction ($V_{m}$) was estimated using the following equation:

$$V_{m}=\frac{I_{178}+I_{188}}{2.2\;I_{262}+I_{178}+I_{188}}$$

where $I_{178}$ and $I_{188}$ represent the intensities (peak heights) of the monoclinic Raman bands at approximately 178 and 188 cm^−1^, respectively, and $I_{262}$ represents the intensity (peak height) of the tetragonal Raman band at approximately 262 cm^−1^. The factor 2.2 is a correction coefficient that accounts for differences in Raman scattering efficiency between the monoclinic and tetragonal phases. The calculated $V_{m}$ value represents the relative monoclinic phase fraction at the surface (Munoz Tabares and Anglada 2010).

### 2.8 Statistical Analysis

The dynamic preloading survival rate was calculated for each group. Fracture resistance in N was analyzed directly, while the bending moment (*M*, N cm) was calculated using the equation M = 5.5 × *F*/10.

To assess the normality of the data distribution, the Shapiro–Wilk test was used in combination with visual inspection of the histogram.

For comparison of load‐to‐fracture outcomes among the three main implant design groups (OPI, BLTPI, and TLTPI; *n* = 18 per group), a one‐way analysis of variance (ANOVA) was performed. A separate one‐way ANOVA was used to compare the TPI subgroups (BLC, BLT, TLC, TLT; *n* = 9 per subgroup). When the overall ANOVA indicated significant differences, post hoc multiple comparisons were conducted as follows:

- Tukey's HSD test when the homogeneity of variances was satisfied.
- Games–Howell test when variance homogeneity was lacking.
- Levene's test was used to evaluate the homogeneity of variances.

To further clarify the influence and interaction of the implant level (bone‐level vs. tissue‐level) and abutment screw material (ceramic vs. titanium) within the TPI subgroups, a two‐way ANOVA was performed, with implant level and screw material specified as fixed factors. No additional post hoc test was performed for the two‐way ANOVA. All analyses were performed using IBM SPSS Statistics (Version 30.0; IBM Corp., Armonk, NY, USA). The level of statistical significance was set at *p* < 0.05 for all tests.

This in vitro study was reported in accordance with the Checklist for Reporting In vitro Studies (CRIS) guidelines (the checklist was provided as a supporting information for the review process).

## 3 Results

### 3.1 Dynamic Preloading Test

All 59 specimens across the main groups and subgroups completed the dynamic preloading tests, with no failures observed.

### 3.2 Static Load‐to‐Fracture Test

After the dynamic preloading test, all surviving specimens were subjected to the static load‐to‐fracture test. Five specimens, one from each group and subgroup, were reserved for crystalline phase evaluation after dynamic preloading and were therefore excluded from the static load‐to‐fracture testing. The one‐way ANOVA showed a statistically significant effect of implant design (OPI, BLTPI, TLTPI) on both the fracture resistance (N) and bending moment (N cm), *F*(2, 51) = 69.80, *p* < 0.001. Post hoc analysis revealed a similar statistically significant pattern (*p* < 0.001), with TLTPI showing the highest values, followed by OPI and BLTPI with the lowest values.

A one‐way ANOVA was conducted to compare the four TPI designs (BLC, BLT, TLC, and TLT). The results demonstrated a significant difference among TPI designs for both fracture resistance (N) and bending moments (N cm), *F*(3, 32) = 38.65, *p* < 0.001. Post hoc analysis revealed that the tissue‐level designs (TLC and TLT) exhibited significantly higher fracture resistance and bending moments than bone‐level designs (BLC and BLT), irrespective of abutment screw material (*p* < 0.001). However, within each implant level, the differences between abutment screw materials (ceramic vs. titanium) were not statistically significant for either the bone‐level designs (BLC vs. BLT designs; *p* = 0.369) or the tissue‐level designs (TLC vs. TLT; *p* = 0.387). Additional details are available in Tables 3, 4a, 4b and Table S1.

**TABLE 3:** Fracture resistance (N) and bending moment (N cm) of each design group and subgroup (Mean ± SD).

Group and subgroup	*n*	Fracture (N) Mean ± SD	Bending moment (N cm) Mean ± SD
OPI	18	605.7 ± 37.4	333.1 ± 20.6
BLTPI	18	498.3 ± 93	274.1 ± 51.2
TLTPI	18	775.2 ± 71	426.4 ± 39
Total	54	626.4 ± 134.5	344.5 ± 74
BLC	9	528.9 ± 101.2	290.9 ± 55.7
BLT	9	467.7 ± 77.7	257.2 ± 42.7
TLC	9	745.2 ± 50.9	409.9 ± 28
TLT	9	805.2 ± 78	442.9 ± 42.9
Total	36	636.8 ± 162.4	350.2 ± 89.3

Abbreviations: BLC, bone‐level two‐piece implant with ceramic abutment screw; BLT, bone‐level two‐piece implant with titanium abutment screw; BLTPI, bone‐level two‐piece implant; *n*, number of specimens; OPI, one‐piece implant; SD, standard deviation; TLC, tissue‐level two‐piece implant with ceramic abutment screw; TLT, tissue‐level two‐piece implant with titanium abutment screw; TLTPI, tissue‐level two‐piece implant.

**TABLE 4a:** Games‐Howell post hoc pairwise comparisons of fracture resistance (N) and bending moment (N cm) among the main implant design groups.

Design groups (1–2)	Mean difference (1–2)	Mean difference (1–2)	*p*	95% confidence interval	
	Fracture (N)	Bending moment (N cm)		Lower–upper (fracture, N)	Lower–upper (bending moment, N cm)
OPI‐BLTPI	107.4*	59.1*	< 0.001	48.1 to 166.7	26.5 to 91.7
OPI‐TLTPI	−169.6*	−93.3*		−216.6 to −122.6	−119.1 to −67.4
BLTPI‐TLTPI	−276.9*	−152.3*		−344.7 to −209.2	−189.6 to −115

*Note:* Games‐Howell post hoc test was used due to unequal variances.

Abbreviations: BLTPI, bone‐level two‐piece implant; OPI, one‐piece implant; TLTPI, tissue‐level two‐piece implant.

* Mean difference significant at *p* < 0.05.

**TABLE 4b:** Tukey post hoc pairwise comparisons of fracture resistance (N) and bending moment (N cm) among the two‐piece implant (TPI) subgroups.

Design groups (1)–(2)	Mean difference (1–2)	Mean difference (1–2)	*p*	95% confidence interval	
	Fracture (N)	Bending moment (N cm)		Lower–upper (fracture, N)	Lower–upper (bending moment, N cm)
BLC‐BLT	61.2	33.6	0.369	−39.6 to 162.1	−21.8 to 89.1
BLC‐TLC	−216.3*	−118.9*	< 0.001	−317.2 to −115.4	−174.4 to −63.5
BLC‐TLT	−276.3*	−151.9*		−377.2 to −175.4	−207.4 to −96.5
BLT‐TLC	−277.5*	−152.6*		−378.4 to −176.6	−208.1 to −97.1
BLT‐TLT	−337.5*	−185.6*		−438.4 to −236.6	−241.1 to −130.1
TLC‐TLT	−60	−33	0.387	−160.8 to 40.8	−88.4 to 22.4

Abbreviations: BLC, bone‐level two‐piece implant with ceramic abutment screw; BLT, bone‐level two‐piece implant with titanium abutment screw; TLC, tissue‐level two‐piece implant with ceramic abutment screw; TLT, tissue‐level two‐piece implant with titanium abutment screw.

* Mean difference significant at *p* < 0.05.

In addition, a two‐way ANOVA was used to assess the effects of both the implant level factor and screw material factor on the TPI designs. The analysis showed a significant main effect of implant level factor (*p* < 0.001), whereas the main effect of screw material was not significant (*p* = 0.982). Further, the interaction between implant level and screw material was significant (*p* = 0.028). For further details, see Table 5.

**TABLE 5:** Two‐way ANOVA of the two‐piece implant (TPI) subgroups evaluating the effect of implant level (bone‐level vs. tissue‐level) and screw material (ceramic vs. titanium) as fixed factors.

Source	dF	*F*	*p*
Implant level	1	110.6	< 0.001*
Screw material	1	0.001	0.982
Implant level × screw material	1	5.30	0.028*

* Statistically significant at *p* < 0.05.

### 3.3 Fracture Mode

All the OPI designs showed fractures in the threaded region of the implant body, specifically at the third and fourth coronal threads, corresponding to the top level of the specimen holder. A similar fracture pattern was also observed in the BLTPI and TLTPI designs. However, in the configurations with ceramic abutment screws, the fracture lines extended slightly more apically, toward the fourth and fifth thread regions. Fractures of the ceramic abutment screw were also observed in BLC and TLC specimens. These fractures occurred in the lower third of the screw, close to the first thread of the abutment screw, and corresponded to the level of the fourth and fifth implant thread fractures. However, in TPI designs with a titanium abutment screw, the fracture lines were observed in a more apical position, within the region of the fifth and sixth threads.

**FIGURE 5:**
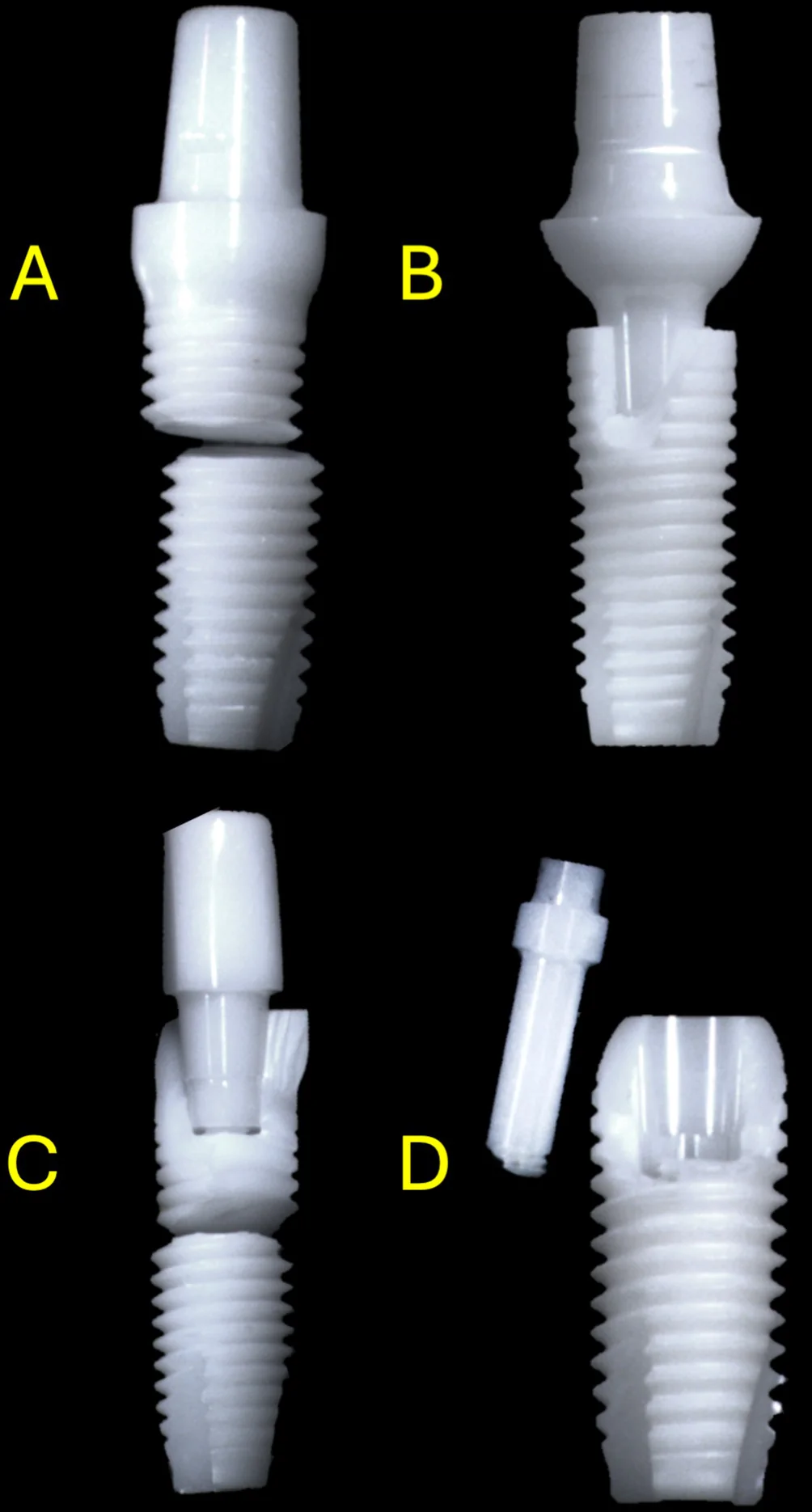
Predominant fracture modes for the evaluated designs. (A) One‐piece implant (OPI): Fracture in the third and fourth thread region. (B) Bone‐level two‐piece implant (BLTPI): Implant platform fracture extends to the third or fourth thread, thereby exposing the abutment. (C) Tissue‐level two‐piece implant (TLTPI): Fracture in the fifth and sixth thread region combined with implant platform fracture extends to the third or fourth thread, thereby exposing the abutment. (D) Ceramic abutment screw: Fracture in the lower third of the abutment screw, close to the first thread, combined with and corresponding to the level of the fourth and fifth implant thread fracture.

The predominant fracture pattern observed in the BLTPI specimens was implant platform fracture, occurring at the interface between the implant and the abutment and extending down to, or just above, the top level of the specimen holder, thereby exposing the abutment. Several specimens exhibited combined fracture patterns, characterized by both implant platform fractures and thread region fracture patterns, which were more common with the TLTPI designs. Across all designs, neither the abutments nor the titanium abutment screws exhibited any fractures. Details regarding fracture modes are available in Figure 5.

### 3.4 SEM Analysis of Fracture Surfaces

**FIGURE 6:**
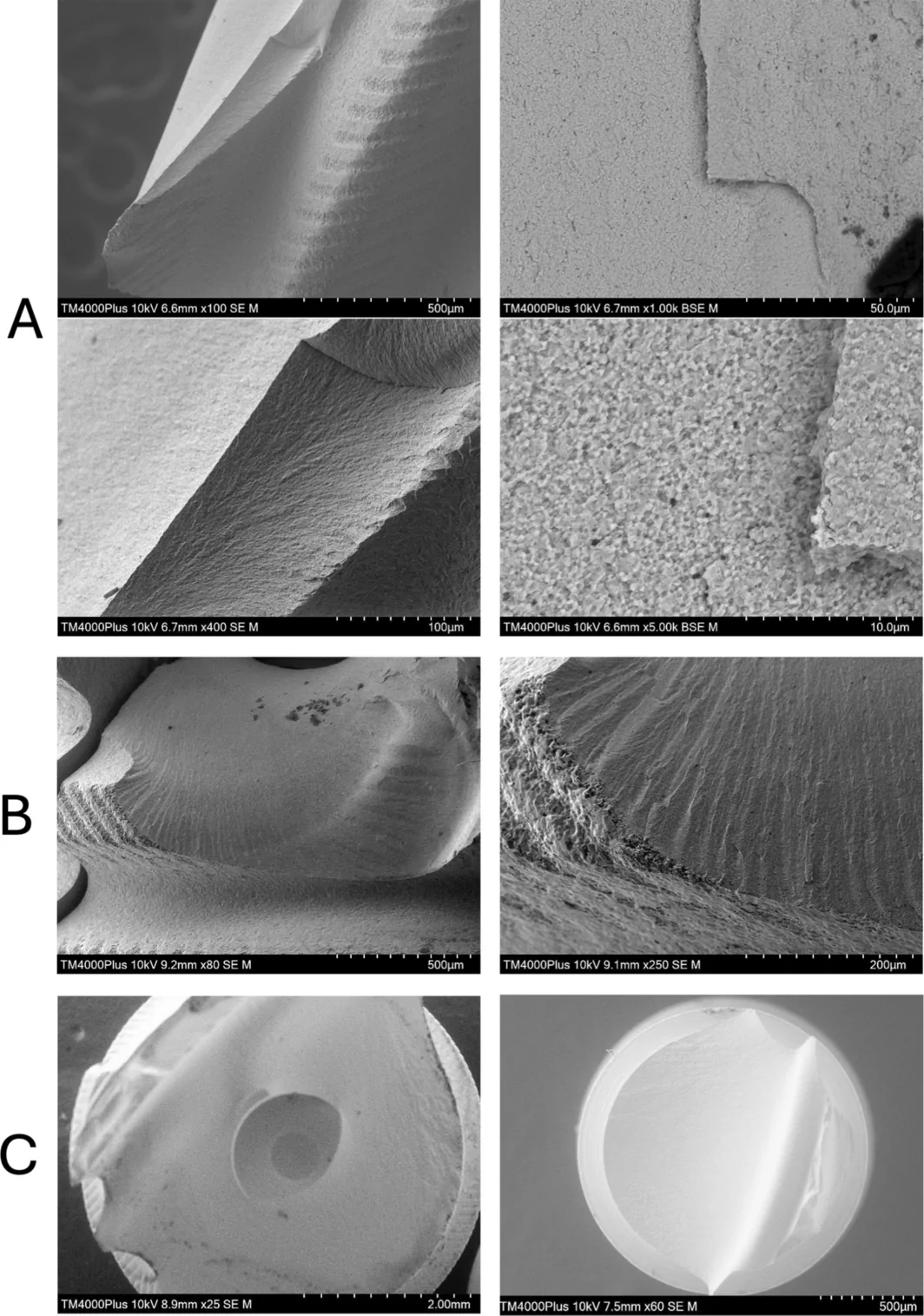
Scanning electron microscopy (SEM) images of surfaces. (A) Images of one‐piece implant (OPI) fracture surfaces in the third and fourth thread region, with four different magnifications. (B) Images of bone‐level two‐piece implant (BLTPI) fracture surfaces in the apical part of the platform fracture, with two different magnifications. (C) Image of tissue‐level two‐piece implant (TLTPI) fracture in the thread region involving the screw chamber or channel (left image). Image of ceramic abutment screw thread fracture (right image).

Representative SEM images of specimens from the investigated implant designs are shown in Figure 6.

After load‐to‐fracture testing, across all main groups (OPI, BLTPI, TLTPI), the fracture surfaces exhibit features characteristic of brittle failure in zirconia. At lower magnification, relatively smooth regions are observed, which can be associated with crack initiation and early propagation. These regions transition into progressively rougher surfaces with increasing topographical variation, indicating crack propagation and final catastrophic failure. At higher magnification, images reveal well‐defined crack paths and localized flat regions, suggesting distinct crack initiation zones. These areas are followed by rougher fracture surfaces with fine‐scale features consistent with transgranular fracture. The fracture morphology appears relatively homogeneous, with no evidence of extensive grain pull‐out or mixed fracture modes.

Similar fracture surface characteristics are observed across the implant designs (OPI, BLTPI, TLTPI), with no distinct differences in fracture morphology between groups.

### 3.5 Crystalline Phases

**FIGURE 7:**
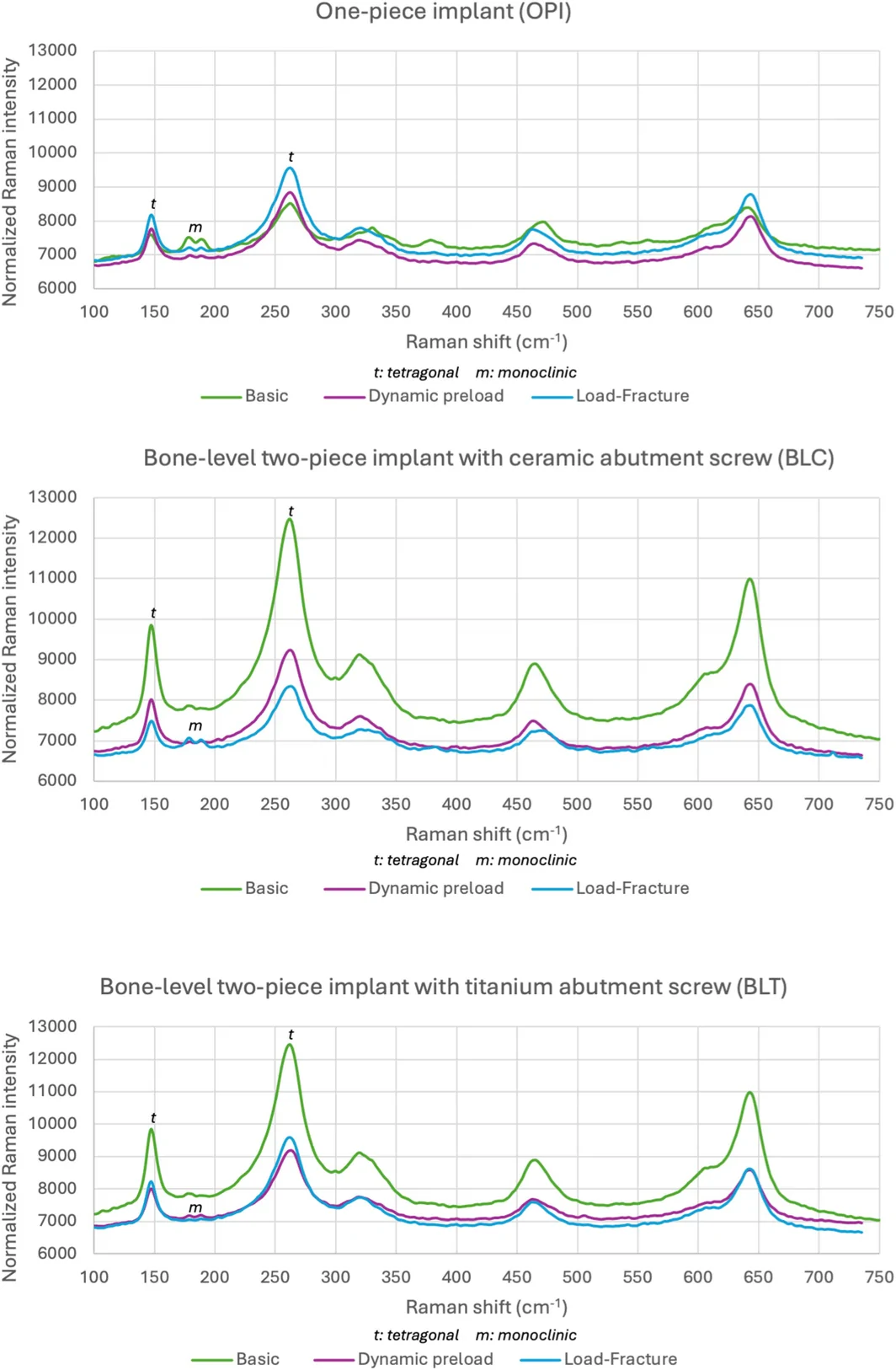
Micro‐Raman spectra of the one‐piece implant (OPI) and bone‐level two‐piece implant (BLTPI) evaluated under baseline conditions and after dynamic preloading and load‐to‐fracture testing. For the OPI and the BLTPI (BLC and BLT), changes in the Raman spectra were observed after mechanical aging and fracture, characterized by an increase in monoclinic zirconia features (*m*) in the low‐wavenumber region (~180–190 cm^−1^), alongside the dominant tetragonal zirconia peaks (*t*) at approximately 250–265 cm^−1^. The TLTPI and the ceramic abutment screw exhibited only tetragonal zirconia peaks in all tested conditions, with no detectable monoclinic phase contribution.

Micro‐Raman spectroscopy indicated that in the basic evaluation, all evaluated implant designs and ceramic abutment screws exhibited tetragonal phase bands. An exception was noted for the one‐piece implant (OPI), in which monoclinic phase bands were already detected under basic conditions. For the OPI design, monoclinic phase bands were also detected following both dynamic preloading and static load‐to‐fracture testing. In contrast, for the BLTPI, only the tetragonal phase was detected in the basic evaluation. Following dynamic preloading, monoclinic phase bands were detected in the BLT design. After the static load‐to‐fracture test, monoclinic phase bands were detected in the BLC design. Additional details are presented in Figure 7.

For the TLTPI design, only tetragonal phase bands were detected in the basic evaluation and after dynamic preloading and static load‐to‐fracture testing. Similarly, the ceramic abutment screws evaluated in both BL and TL designs showed only tetragonal phase bands at all evaluation stages.

Regarding the monoclinic phase fraction (*V*_*m*_), across all the investigated specimens, there was a limited increase in the monoclinic phase of approximately 1%–2% following dynamic preloading and load‐to‐fracture testing, compared to the basic or before testing evaluations. With the exception of the OPI design, which demonstrated a higher monoclinic fraction during the basic evaluation and a lower monoclinic fraction in the specimens evaluated after testing. Another exception is the BLC design, which exhibited a higher monoclinic fraction after the load‐to‐fracture test. Summary of the Micro‐Raman‐based estimation of the monoclinic phase fraction (*V*_*m*_) and band shifts before testing (basic), after dynamic preload, and after load‐to‐fracture testing across all investigated specimens is shown in Table S2.

## 4 Discussion

Fracture of ceramic implants was a major concern previously; after that aluminum oxide implants were withdrawn from the market due to insufficient mechanical properties (Andreiotelli et al. 2009). Although early studies reported relatively few fractures of zirconia implants, many early systems were later discontinued due to design issues, such as small implant diameters or surface roughness, or because of manufacturing‐related flaws (Almarghlani and Alshali 2022; Gahlert et al. 2013; Roehling et al. 2016). Recent advances in zirconia materials and implant design have decreased fracture risk (Bazal‐Bonelli et al. 2025; Mohseni et al. 2023; Roehling et al. 2018). Nevertheless, the German Society for Implantology (DGI) and the European Association for Osseointegration (EAO) recommend further investigation of zirconia TPI (Balmer et al. 2022; Thiem et al. 2022). To our knowledge, this is the first in vitro study to investigate commercially available screw‐retained zirconia implants, specifically both BLTPI and TLTPI configurations equipped with ceramic abutment screws, within the same study.

All investigated groups and subgroups survived 2 million cycles of dynamic preloading while immersed in 37°C distilled water, which approximates intraoral temperature. This indicates that the specimens showed adequate resistance to dynamic preloading under the applied aging conditions. In a previous study that used the BLTPI implant, one specimen failed during dynamic loading; however, that study used a more rigorous protocol of 10 million cycles in a 5°C–55°C thermocycling container (Kohal et al. 2024). At present, no consensus exists regarding standardized recommendations for the number of loading cycles to be used in in vitro testing. A systematic review of 19 in vitro studies on the fracture strength of zirconia implants reported that the number of applied cycles ranged from 1.2 million to 10 million (Bethke et al. 2020). It has been suggested that 10 million cycles correspond to approximately 12.5–40 years of clinical lifetime (Kohal et al. 2024; Rosentritt et al. 2006). Accordingly, the 2 million cycles used in this study correspond to approximately 2.5–8 years of simulated clinical service.

Regarding the load‐to‐fracture results, the TLTPI design demonstrated significantly higher fracture resistance (775 ± 71 N) and bending moments (426 ± 39 N cm) than both the OPI and the BLTPI designs (*p* < 0.001). This superior performance might be related to the two‐piece configuration of the TLTPI system, in which the abutment and screw collectively share and distribute mechanical stresses under loading. Moreover, the implant platform in the connection area is well supported by a tissue collar, allowing for better stress distribution compared to OPI designs, where stresses are concentrated in one region (Avağ and Akkocaoğlu 2023; Kim et al. 2020).

Bethke et al. (2020) reported a higher bending moment for the OPI design than for TPI designs, which contrasts with our results regarding TLTPI configuration. However, their results are consistent with our comparison between OPI and BLTPI designs. In our study, the OPI demonstrated significantly higher fracture strength (606 ± 37 N) and bending moment (333 ± 21 N cm) compared to the BLTPI design (fracture resistance: 498 ± 93 N; bending moment: 274 ± 51 N cm). Compared with OPI, the BLTPI design features a more complex mechanical design of the implant‐abutment connection area, which may introduce a weakness at the implant platform edge when pressed by the abutment during loading (Avağ and Akkocaoğlu 2023; Kim et al. 2020). Although BLTPI designs exhibited lower fracture resistance than both the TLTPI and the OPI designs, the reported fracture resistance remained above 200 N cm, which is considered adequate for clinical safety (Bethke et al. 2020; Kohal et al. 2024; Mohseni et al. 2023). Thus, the implant design influences the fracture strength and bending moments of zirconia implants. As a result, the first null hypothesis—which stated that there is no difference in the fracture resistance and fracture mode between one‐piece and bone‐level or tissue‐level two‐piece zirconia dental implant designs after aging by dynamic preloading in a moist environment—was rejected.

For the evaluated TPI subgroups (BLC, BLT, TLC, and TLT), significant differences were observed between implant levels (BL vs. TL). In contrast, designs within the same implant level, regardless of abutment screw type (ceramic or titanium), showed no significant differences. These findings were consistent with the two‐way ANOVA results, which showed a significant effect of implant level but not of screw material. Although not statistically significant, the tissue‐level implants showed slightly higher mean values when paired with the titanium abutment screws, whereas the bone‐level implants showed slightly higher mean values when paired with the ceramic abutment screws. Therefore, the interaction between implant level and screw material was significant (*p* = 0.028), indicating that the effect of the screw material on the fracture strength and bending moment differed between bone‐level and tissue‐level implants. The order of fracture resistance and bending moment, from highest to lowest, was TLT, TLC, BLC, and BLT.

These results are in line with the report by Kohal et al., which found no difference between the BL zirconia implants with ceramic abutment screws and titanium abutment screws (Kohal et al. 2024). Although their specimens underwent 10 million loading cycles, the mean fracture and bending moment values reported for BLC (826 ± 114 N; 456 ± 62 N cm) and BLT (652 ± 256 N; 356 ± 139 N cm) were higher than those observed in the present study for BLC (529 ± 101 N; 291 ± 55 N cm) and for BLT (468 ± 77 N; 257 ± 43 N cm). These differences may be explained by methodological variations. For instance, in the present study, the lower border of the loading hemisphere was positioned on the abutment margin, whereas in the previous study, the loading hemisphere ended short of this point. Additionally, differences in the abutment design and specimen holder configuration may have influenced both the magnitude and direction of the load applied to the implant‐abutment complex. Furthermore, the crosshead speed used for load‐to‐fracture testing in the aforementioned study was 10 mm/min, compared with 0.5 mm/min in the present study, which might be more challenging for brittle materials (Quinn 2007). A previous systematic review also reported that crosshead speed significantly affected TPI designs (Bethke et al. 2020).

A recently published clinical study evaluated zirconia TL and BL designs with a ceramic abutment screw and reported that both groups survived the 2‐year follow‐up period with no failures, except for one ceramic abutment screw that fractured during the screw tightening process (Žarković Gjurin et al. 2025). To date, no in vitro studies have compared the TL zirconia implant with ceramic (zirconia) and titanium abutment screws.

Accordingly, the second null hypothesis—stating that there is no difference in fracture resistance or fracture mode following dynamic preloading in a moist environment between bone‐level and tissue‐level designs of two‐piece zirconia implants, nor between designs with abutment screws made of ceramic versus titanium. – was partially rejected.

The main implant fracture modes observed in this study included: (1) fractures occurring in the threaded region of the implant at the third and fourth coronal threads; (2) fractures in a similar region but extending slightly more apically toward the fourth and fifth threads, or further apically in the fifth and sixth threads; and (3) implant platform fractures, occurring at the interface between the implant and the abutment, extending down to the top level of the specimen holder or just above it, thereby exposing the abutment. The OPI design showed only fractures in the threaded region of the implant, specifically at the third and fourth coronal threads. In contrast, the BLTPI design most commonly showed implant platform fractures, with a few cases showing thread‐level fractures involving the fourth and fifth threads, or the fifth and sixth threads. The TLTPI design showed fractures involving both the implant platform and the implant thread region, typically affecting the fourth and fifth threads, or in some cases, the fifth and sixth threads. In a few cases, only one fracture pattern was observed. These fracture patterns are consistent with those reported in a study analyzing clinically fractured zirconia implants, which coincide with those reported in the present study (Zhang et al. 2022). In addition, an in vitro study reported similar fracture patterns for OPI and BLTPI designs (Burkhardt et al. 2021). The thread fractures observed in the fifth and sixth thread regions were commonly associated with the titanium abutment screw designs, thereby a titanium screw fracture.

A ceramic screw fracture was also observed and corresponds to a thread‐region fracture involving the fourth and fifth threads. Furthermore, no abutment fractures were observed in this study, in contrast to clinical reports that document cases of abutment fractures with zirconia implants (Brunello et al. 2022; Cionca et al. 2021). It is important to note, however, that those clinical cases involved different implant systems and were subjected to a variety of loading conditions that differ from the controlled in vitro environment.

SEM observations indicate that fractures in the investigated groups occur through typical brittle mechanisms of dense yttria‐stabilized zirconia. The presence of smooth regions associated with crack initiation, followed by rougher propagation zones, indicates failure dominated by a single critical flaw and subsequent crack growth under increasing stress. A comparable brittle fracture was reported in a study analyzing fractured zirconia implants (Zhang et al. 2022). The absence of distinct differences in fracture surface morphology between OPI, BLTPI, and TLTPI configurations indicates that the underlying fracture mechanism is not significantly altered by implant design. Instead, the fracture mechanism appears to be governed primarily by local stress concentrations and inherent flaw populations.

Regarding the evaluation of crystalline phases, the zirconia material remained predominantly tetragonal after dynamic preloading and load‐to‐fracture testing. Nevertheless, localized tetragonal‐to‐monoclinic (*t* → *m*) transformation was detected at specific measurement locations. Only the OPI designs showed a detectable monoclinic band in the basic evaluation, after dynamic preloading, and after static load‐to‐fracture testing. The presence of the monoclinic band in the basic evaluation and the subsequent decrease in the monoclinic fraction in later evaluations may be attributable to the fact that different specimens were used in each evaluation, and the specimen used in the basic evaluation had a slightly higher monoclinic fraction. Kohal et al. (2024) and Burkhardt et al. (2021) also reported the presence of the monoclinic phase prior to testing. However, their observations were made in BLTPI implant designs. In the present study, no monoclinic band was observed in the BLTPI design during the basic evaluation. The monoclinic band transformation was observed after dynamic preloading and following static load‐to‐fracture testing. The TLTPI design and the evaluated ceramic screw both showed a tetragonal band in all evaluations.

The results of the monoclinic phase fraction generally indicated a low amount of monoclinic phase, with the material remaining mainly tetragonal across all designs following testing. In the case of BLC, after the load‐to‐fracture test and basic evaluation of the OPI designs, a higher amount of monoclinic fraction was detected compared to other designs. Overall, the micro‐Raman results suggest that implant design and loading history influence susceptibility to surface phase transformation. However, the zirconia material evaluated in this study remained predominantly tetragonal throughout.

The Micro‐Raman findings should be interpreted with caution and should be interpreted comparatively rather than as representative of bulk phase composition. Given the surface‐sensitive and semi‐quantitative nature of Micro‐Raman spectroscopy, along with the limited number of evaluated specimens and locations. Nevertheless, the consistent location‐ and load‐dependent trends observed in this study support the utility of micro‐Raman spectroscopy for assessing stress‐induced phase transformations in zirconia implant systems. Accordingly, the third hypothesis—stating that crystalline phases of zirconia implants are not influenced by aging (dynamic preloading in a moist environment) or by fracture testing, regardless of implant design—is partially rejected.

Overall, the present study has limitations associated with its in vitro design. Such an approach cannot fully capture the complexity of clinical reality, and although efforts were made to simulate the oral environment, these methods do not ensure that the tested specimens accurately represent clinical setting performance. Additional limitations include the limited number of cycles (2 million) and the use of a stable moist temperature instead of thermocycling. Therefore, the findings should be interpreted as an approximation of clinical behavior and require further validation through future clinical studies. Regarding sample size, the power analysis was done for the three main design groups (OPI, BLTPI, TLTPI). However, for the TPI subgroups (BLC, BLT, TLC, TLT), nine specimens were allocated for each subgroup. This number was sufficient to detect significant differences between the implant‐level designs (BL and TL). However, for assessing the influence of the abutment screw design/material, small differences may have gone undetected with the current sample size.

## 5 Conclusion

Within the limitations of this in vitro study, the results suggest that the evaluated zirconia‐based OPI, BLTPI, and TLTPI systems might provide sufficient fracture resistance to support further clinical investigation. The implant‐level design (TL vs. BL) influences mechanical performance, whereas the influence of abutment screw material (ceramic vs. titanium) is less pronounced and varies according to the implant‐level design. From a mechanical perspective, the TLTPI design appears to be the most favorable option, followed by the OPI design, with the BLTPI design being the least advantageous.

Distinct fracture modes were associated with each design: fractures in the OPI occurring primarily at the coronal thread region, platform fractures predominating in the BLTPI design, and combined thread‐region and platform fractures observed in the TLTPI design. Micro‐Raman analysis indicated that the evaluated zirconia material remained predominantly tetragonal, with localized monoclinic phase formation depending on implant design and loading history.

Further prospective clinical studies with mid‐ and long‐term follow‐up are required to confirm these findings and to assess the clinical performance of the different zirconia implant designs under functional loading.

## Author Contributions

**Evaggelia Papia:** conceptualization, methodology, validation, supervision, project administration, writing – review and editing, investigation, formal analysis, resources, data curation. **Magnus Anderson:** investigation, methodology, data curation, validation, visualization, writing – review and editing. **Siamak Eqtesadi:** methodology, investigation, validation, formal analysis, visualization, writing – review and editing. **Jonas P. Becktor:** conceptualization, methodology, supervision, validation, writing – review and editing, resources. **Per Vult von Steyern:** conceptualization, methodology, validation, investigation, supervision, project administration, writing – review and editing, resources. **Abdulaziz Gul:** conceptualization, methodology, data curation, investigation, validation, formal analysis, writing – review and editing, writing – original draft, visualization, project administration, resources.

## Funding

This study was supported by the Knowledge Foundation grant number 20250008 to the Advancing Oral Health research profile. Z‐Systems AG (Oensingen, Switzerland) supplied the study materials, including implants, abutments, ceramic and titanium abutment screws, and screwdrivers. Z‐Systems AG's involvement was limited to providing materials, with no additional financial support received.

## Ethics Statement

The authors have nothing to report.

## Consent

The authors have nothing to report.

## Conflicts of Interest

The authors declare that they have no conflicts of interest. The funder and the material provider were not involved in the study design, data collection, analysis, interpretation, manuscript preparation, or publication.

## Supporting information


**Table S1:** One‐way ANOVA results for fracture resistance (N) and bending moment (N cm) of the main implant groups and TPI subgroups.

**Table S2:** Summarize the Micro‐Raman‐based estimation of the monoclinic phase fraction (*V*_*m*_) and band shifts before testing (basic), after dynamic preload, and after load‐to‐fracture testing across all investigated specimens.

## Data Availability

The datasets used and/or analyzed during the current study are available from the corresponding author on reasonable request.
