# Supplementary material for: Effect of Implant Design on Fracture Resistance, Mode, and Crystal Structure of One‐ and Two‐Piece Zirconia Implants With Ceramic or Titanium Abutment Screws: An In Vitro Study
Source: Clin Oral Implants Res. 2026 Jun 13;37(9):1081–95. doi: 10.1111/clr.70149 (PMC13542745; doi:10.1111/clr.70149)
Supplement: Supplementary file 1 [file CLR-37-1081-s001.docx]

**Appendices**

**Supplementary Table S1.** One-way ANOVA results for fracture resistance (N) and bending moment (Ncm) of the main implant groups and TPI subgroups.

| **Group comparison** | **Source of variation** | **dF** | **F** | **P-value** |
| --- | --- | --- | --- | --- |
| **Main groups (OPI, BLTPI and TLTPI)** | Between groups | 2 | 69.80 | *< 0.001** |
|  | Within groups | 51 | - | - |
| **TPI subgroups (BLC, BLT, TLC and TLT)** | Between groups | 3 | 38.65 | *< 0.001** |
|  | Within groups | 32 | - | - |

Abbreviations: ANOVA: analysis of variance; df: degree of freedom; F: variance ratio; OPI: one-piece implant; BLTPI: bone-level two-piece implant; TLTPI: tissue-level two-piece implant; BLC: bone-level two-piece implant with ceramic abutment screw; BLT: bone-level two-piece implant with titanium abutment screw; TLC: tissue-level two-piece implant with ceramic abutment screw; TLT: tissue-level two-piece implant with titanium abutment screw.

*Statistically significant at *p<0.05*.
