# Supplementary material for: Effect of Implant Design on Fracture Resistance, Mode, and Crystal Structure of One‐ and Two‐Piece Zirconia Implants With Ceramic or Titanium Abutment Screws: An In Vitro Study
Source: Clin Oral Implants Res. 2026 Jun 13;37(9):1081–95. doi: 10.1111/clr.70149 (PMC13542745; doi:10.1111/clr.70149)
Supplement: Supplementary file 2 [file CLR-37-1081-s002.docx]

**Appendices**

**Supplementary table S2.** Summarize the Micro-Raman-based estimation of the monoclinic phase fraction (V*m*) and band shifts before testing (basic), after dynamic preload, and after load-to-fracture testing across all investigated specimens.

| **Group** | **Evaluation stage** | **V*m* (Katagiri, %)** | **V*t** = (*t+c*) (%)** | **Main band positions (cm⁻¹)** | **Band shifts Δν (cm⁻¹)** |
| --- | --- | --- | --- | --- | --- |
| **OPI** | Basic | ≈ 13-14 | ≈ 86–87 | 178.6 / 189 / 262.7 / 641.7 | +0.6 / +1.0 / +0.7 / +1.7 |
|  | Dynamic preloading | ≈3-4 | ≈96-97 | 179.3 / 189.0 / 262.0 / 643.7 | +1.3 / +1.0 / +0.0 / +3.7 |
|  | Load-to-fracture | ≈ 5–6 | ≈ 94–95 | 179.4 / 188.4 / 262.7 / 642.7 | +1.4 / +0.4 / +0.7 / +2.7 |
| **BLTPI** | Basic | ≈1–2 | ≈98–99 | 177.9 / 188.3 / 262.0 / 642.4 | −0.1 / +0.3 / +0.0 / +2.4 |
| **BLC** | Dynamic preloading | ≈2 | ≈98 | 179.3 / 188.3 / 262.7 / 643.0 | +1.3 / +0.3 / +0.7 / +3.0 |
|  | Load-to-fracture | ≈8-9 | ≈91-92 | 178.6 / 188.3 / 262.7 / 643.0 | +0.6 / +0.3 / +0.7 / +3.0 |
| **BLT** | Dynamic preloading | ≈ 4 | ≈ 96 | 178.7 / 188.4 / 262.7 / 642.7 | +0.7 / +0.4 / +0.7 / +2.7 |
|  | Load-to-fracture | ≈ 1–2 | ≈ 98–99 | 179.4 / 188.4 / 262.0 / 642.1 | +1.4 / +0.4 / +0.0 / +2.1 |
| **TLTPI** | Basic | ≈2 | ≈98 | 177.9 / 190.4 / 262.0 / 641.7 | -0.1 / +2.4 / +0.0 / +1.7 |
| **TLC** | Dynamic preloading | ≈3 | ≈97 | 179.3 / 188.3 / 261.3 / 643.0 | +1.3 / +0.3 / -0.7 / +3.0 |
|  | Load-to-fracture | ≈3 | ≈97 | 179.3 / 188.3 / 262.7 / 643.0 | +1.3 / +0.3 / +0.7 / +3.0 |
| **TLT** | Dynamic preloading | ≈2 | ≈98 | 178.6 / 188.3 / 262.7 / 643.0 | +0.6 / +0.3 / +0.7 / +3.0 |
|  | Load-to-fracture | ≈3 | ≈97 | 178.6 / 189.0 / 262.0 / 643.0 | +0.6 / +1.0 / +0.0 / +3.0 |
| **Ceramic abutment screw** | Basic | ≈0–1 | ≈99–100 | 177.2 / 191.8 / 262.0 / 643.0 | −0.8 / +3.8 / +0.0 / +3.0 |
| **Ceramic abutment screw in BLTPI** | Dynamic preloading | ≈3 | ≈97 | 179.3 / 188.3 / 262.0 / 643.7 | +1.3 / +0.3 / +0.0 / +3.7 |
|  | Load-to-fracture | ≈3 | ≈97 | 179.3 / 188.3 / 262.0 / 643.0 | +1.3 / +0.3 / +0.0 / +3.0 |
| **Ceramic abutment screw in TLTPI** | Dynamic preloading | ≈3 | ≈97 | 178.6 / 188.3 / 262.0 / 643.0 | +0.6 / +0.3 / +0.0 / +3.0 |
|  | Load-to-fracture | ≈4 | ≈96 | 179.3 / 188.3 / 261.3 / 643.7 | +1.3 / +0.3 / −0.7 / +3.7 |

V*m* was estimated using the Katagiri equation based on baseline-corrected monoclinic peak heights at approximately 178 and 188 cm⁻¹ and the tetragonal peak height at approximately 262 cm⁻¹. V*t** was calculated as 100 - V*m* and represents the combined tetragonal (*t)* and cubic (*C*) fraction. The cubic phase was not quantified separately because its Raman bands overlap with the tetragonal phase. The main band positions (cm⁻¹) are the measured positions of the characteristic monoclinic, tetragonal, and mixed bands. Band shifts were calculated relative to the reference band positions of 178, 188, 262, and 640 cm⁻¹. Values are presented as approximate ranges because peak-height estimation depends on baseline selection and local valley-to-valley correction. OPI: one-piece implant; BLTPI: bone-level two-piece implant; TLTPI: tissue-level two-piece implant; BLC: bone-level with a ceramic screw; BLT: bone-level with a titanium screw; TLC: tissue-level with a ceramic screw; TLT: tissue-level with a titanium screw.
